# Explainable hypoglycemia prediction models through dynamic structured grammatical evolution

**DOI:** 10.1038/s41598-024-63187-5

**Published:** 2024-06-01

**Authors:** Marina De La Cruz, Oscar Garnica, Carlos Cervigon, Jose Manuel Velasco, J. Ignacio Hidalgo

**Affiliations:** 1 Universidad Complutense de Madrid, Calle Prof. José García Santesmases,9, Madrid, 28040 Spain; 2 Instituto de Tecnología del Conocimiento, Street, Madrid, Spain

**Keywords:** Diabetes, Hypoglycemia prediction, Rule system, Structured grammatical evolution, Computational biology and bioinformatics, Endocrinology, Mathematics and computing

## Abstract

Effective blood glucose management is crucial for people with diabetes to avoid acute complications. Predicting extreme values accurately and in a timely manner is of vital importance to them. People with diabetes are particularly concerned about suffering a hypoglycemia (low value) event and, moreover, that the event will be prolonged in time. It is crucial to predict hyperglycemia (high value) and hypoglycemia events that may cause health damages in the short term and potential permanent damages in the long term. This paper describes our research on predicting hypoglycemia events at 30, 60, 90, and 120 minutes using machine learning methods. We propose using structured Grammatical Evolution and dynamic structured Grammatical Evolution to produce interpretable mathematical expressions that predict a hypoglycemia event. Our proposal generates white-box models induced by a grammar based on if-then-else conditions using blood glucose, heart rate, number of steps, and burned calories as the inputs for the machine learning technique. We apply these techniques to create three types of models: individualized, cluster, and population-based. They all are then compared with the predictions of eleven machine learning techniques. We apply these techniques to a dataset of 24 real patients of the Hospital Universitario Principe de Asturias, Madrid, Spain. The resulting models, presented as if-then-else statements that incorporate numeric, relational, and logical operations between variables and constants, are inherently interpretable. The True Positive Rate and True Negative Rate metrics are above 0.90 for 30-minute predictions, 0.80 for 60 min, and 0.70 for 90 min and 120 min for the three types of models. Individualized models exhibit the best metrics, while cluster and population-based models perform similarly. Structured and dynamic structured grammatical evolution techniques perform similarly for all forecasting horizons. Regarding the comparison of different machine learning techniques, on the shorter forecasting horizons, our proposals have a high probability of winning, a probability that diminishes on the longer time horizons. Structured grammatical evolution provides advanced forecasting models that facilitate model explanation, modification, and retesting, offering flexibility for refining solutions post-creation and a deeper understanding of blood glucose behavior. These models have been integrated into the glUCModel application, designed to serve people with diabetes.

## Introduction

The progression of diabetes mellitus in recent years has made it one of the most relevant diseases of the 21st century^1^. According to World Health Organization estimations, diabetes will be among the leading causes of death worldwide by 2030. Type 1 diabetes (T1DM) is an autoimmune disease that destroys the insulin-producing cells (beta cells) of the pancreas. A healthy pancreas is responsible for regulating blood glucose levels by producing insulin. Thanks to insulin, the cells of the body can absorb glucose from the bloodstream. For people with diabetes (PwD), the absence of insulin prevents glucose assimilation, causing an increase in glucose levels in the bloodstream. As a result, the person needs to inject insulin to maintain these values in a healthy range. Besides insulin, there are a lot of external factors that affect the variability of blood glucose levels. As a result, it is widespread for PwD to suffer from frequent extreme values provoking hypoglycemia (low values) and hyperglycemia (high values) events, even if the amount of insulin injected is within their usual range. In order to avoid acute and long-term complications, PwD patients have to maintain the glucose levels in their blood within healthy ranges, and they have to pay special attention to hypoglycemia and hyperglycemia.

Maintaining glucose in desired ranges could be very difficult. For instance, physicians recommend that PwD perform moderate regular exercise as it can be a helpful factor in stabilizing blood glucose values and preventing high glucose variability. However, doing exercise also affects insulin sensitivity and can cause hypoglycemia. Reasonable control of the body weight and high insulin sensitivity will reduce the amount of insulin a patient needs to inject per meal and, consequently, the reaction in their blood sugar levels, leading to more stable values. However, the increase in insulin sensitivity and the fact that the muscles need sugar while exercising can result in a state of hypoglycemia, which can be dangerous if it is not appropriately addressed.

The symptoms of hypoglycemia vary depending on the person but usually encompass shaking, sweating, hunger, fast or irregular heartbeat, numbness in extremities, etc. These symptoms can be easily confused while exercising, and a person with diabetes might not be aware that she/he is suffering a hypoglycemia event until it is too late and they enter severe hypoglycemia. How distressing the symptoms become can lead people to actively try having a glucose value higher than recommended levels or even not doing exercise out of fear of entering into a hypoglycemic state. On the other hand, if a patient suffers from recurrent events of hypoglycemia, their body can become used to constantly staying in this state, and the person might lose the ability to recognize a hypoglycemic event. If this is the case, then the blood sugar could drop to shallow values, ending with a loss of consciousness and needing medical attention.

The way to deal with a hypoglycemic state is to ingest a certain amount of carbohydrates (usually 15 g) and wait around 15 min. If the glucose levels have not returned to normal, the process is repeated. This means that by the time a person has entered hypoglycemia, they will stay in this state until the glucose ingested has been absorbed by the body.

Due to the importance of hypoglycemic events in the health of a diabetic person, a variety of techniques have been used to forecast them with different input variables. In this section, we review the state-of-the-art related to our problem. An extensive list can be found in Mujahid et al.^2^.

Most studies use previous glucose values, either alone or paired with other input variables (such as insulin and carbohydrate data, exercise data, breath samples, etc). The forecast horizon also differs from short-term predictions (30 to 60 min) to a longer period of time (240 min to 6 h). A variety of ML models has been used to perform the forecast, including Artificial Neural networks (ANN), Decision Trees (DT), Random Forest (RF), Regression, Kernel, Reinforcement Learning (RL), and Estimating Probability Distribution.

ANNs have been successfully applied to prediction in a wide variety of problems, including hypoglycemia. The study by Bertachi et al.^3^ uses a multilayer perceptron (MLP) to predict nocturnal hypoglycemia events using glucose data from a CGM monitor and data from an activity tracker. The results obtained yield 0.70 and 0.79 of sensitivity and specificity, respectively. Vehi et al.^4^ also perform nocturnal hypoglycemic prediction using glucose information, insulin, carbohydrates, and exercise information on an MLP that makes the classification. Other similar works are San et al.^5^, (Type 1 diabetic children), and Bertachi et al.^6^.

Other authors are focused on hypoglycemic prediction on short-time horizons, such as Mosquera-Lopez et al.^7^ and Mhaskar et al.^8^, that use Recurrent Neural Networks and Deep Neural Networks respectively to predict hypoglycemic and hyperglycemic events on a 30 min time frame.

Support vector machine (SVM) is frequently used to perform forecasting classification. Using SVM, several studies are focused on the prediction of nocturnal hypoglycemia. These include Mosquera-Lopez et al.^9^ that obtained a 0.94 recall of hypoglycemic events, and Güemes et al.^10^, where different techniques were considered and SVM yielded the best results on a 5 h and 18 h time-frame. Other studies perform the prediction of postprandial hypoglycemic episodes, Vehi et al.^4^ and Oviedo et al.^11^ within 240 min after a meal.

DTs are a classification technique with high interpretability that have been used for studying hypoglycemia prediction. Some studies use standard DTs to perform the classification, such as Reddy et al.^12^, where DTs predict hypoglycemic episodes in the context of aerobic exercise. This study also compares the efficiency of DTs with RF. RF is an ensemble of DTs in which the classification is performed through a voting system. RF usually reaches better results at the expense of the interpretability of the model. Reddy et al.’s results show a 0.80 accuracy with DT and two variables and a 0.87 accuracy with RF.

RF is the most used ensemble method for hypoglycemic prediction. It has been used in a lot of studies together with other ML techniques, and comparing its efficiency with other ML models yields outstanding results. Some studies that use RF focus on nocturnal hypoglycemias, Güemes et al.^10^. Other studies try to forecast postprandial hypoglycemia, Seo et al.^13^ with 30 min predictions after a meal. In terms of all-day round predictions on a short-time horizon, the study by Dave et al.^14^ uses RF to perform predictions on a 30 min and 60 min time horizon, returning values of Recall of 0.90 during the day and 0.94 at night.

In summary, many studies perform hypoglycemic classification using different input variables, time horizons, and models. However, most studies choose to predict at specific points in glucose development, such as nocturnal or postprandial predictions, and most of the methods yield black-box models. Moreover, most of them need information about the previous insulin dose administration and carbohydrate ingestion.

The motivation for this research lies in the need for white-box reliable predictive models of low blood glucose levels for people with diabetes. Predicting a future hypoglycemic episode may lead PwD to take remedial action before it occurs and to make better decisions in the future to avoid acute complications. With this motivation as the ultimate goal, in this paper, we investigate the performance of Grammatical Evolution (GE) to obtain classification white-box models for hypoglycemia prediction using glucose values and other variables easily accessible from smartwatch data. The models are composed of a set of if-then-else conditions that evaluate values of a set of physiological variables during the last two hours before the prediction. The conditions to be evaluated include information on a set of physiological variables during the last two hours before the prediction. In particular, we include glucose levels measured by a Continuous Glucose Monitoring System (CGM), heart rate values, number of steps, and calories burned.

As a result, we get explainable expressions that predict a class that represents the future state of the person (hypoglycemia or non-hypoglycemia) in the prediction horizons of 30, 60, 90, and 120 min. We investigate the performance of both Structured Grammatical Evolution (SGE)^15^ and Dynamic Structured Grammatical Evolution (DSGE)^16^. Experimental results with a set of data of patients from a Public Hospital in Spain show that our proposals can produce very good rule-based models.

In addition, our predictive model has been integrated into the web and mobile application known as glUCModel, as previously described in the works^17,18^. This established application is dedicated to serving individuals with diabetes. Notably, the hallmark of this application is its unwavering commitment to user-centric design, offering an intuitive and user-friendly interface that serves as a vital link between PwD and healthcare professionals. Through the glUCModel interface, PwD gains direct access to personalized health information, comprehensive reports on their glucose dynamics, valuable insights derived from this model and other state-of-the-art artificial intelligence algorithms, and input from healthcare experts. This fusion of technology and medical expertise will help individuals proactively manage their condition, make informed decisions, and take timely actions to maintain optimal glucose levels and overall health.

The rest of the paper is organized as follows. Section "Methods and materials" introduces the GE techniques, the data collected from real patients, the three types of models we propose, and the experimental setup. Section "Results" presents the experimental results regarding the clustering of patients, the results of the three types of predictive models, and the comparison with the eleven machine learning algorithms. Section "Discussion" discusses our results in comparison with the state-of-the-art related to our problem, highlights the interpretability of the results, and presents the limitations of this work. Finally, Section "Conclusion" exposes conclusions and future work.

## Methods and materials

In this paper, we investigate the use of Structured Grammatical Evolution (SGE) and Dynamic Structured Grammatical Evolution (DSGE) to obtain prediction models for hypoglycemia events. We devote this section to the details of the methodology, proposed grammar, data, and experimental setup of our experiments.

We propose using Structured Grammatical Evolution (SGE) to obtain a forecasting model to predict future hypoglycemic events. Resulting models are mathematical expressions that, given the input data, predict whether a hypoglycemic event will happen at a certain time horizon.

The input variables are:

- The glucose values for the two hours previous to the prediction time, *t*, sampled every 5 min. We define a variable for each of the 24 sample times in the 2-hour time window, $\texttt {gluc}_s(t)\equiv \texttt {gluc}(t-s)$ with $s \in \{5, 10, 15, \ldots , 120\}$ min.
- Heart rate, hr, for the two hours previous to the prediction time, *t*, sampled every 5 min, $\texttt {hr}_s(t)\equiv \texttt {hr}(t-s)$.
- Number of steps, steps, for the two hours previous to the prediction time, *t*, sampled every 5 min, $\texttt {steps}_s(t) \equiv \texttt {steps}(t-s)$.
- Calories burned, cal, for the two hours previous to the prediction time, *t*, sampled every 5 min, $\texttt {cal}_s(t) \equiv \texttt {cal}(t-s)$.

The glucose measurements were obtained using a CGM system, and the exercise-related data were collected using a Fitbit Ionic smartwatch.

The output variable is the expected class, $\texttt {hyp}$, at the prediction horizon, ph; i.e., ph minutes after the prediction time. We predict at four prediction horizons: 30 min, 60 min, 90 min, and 120 min. We denote these values as $\texttt {hyp}(t+\texttt {ph})$ with ph$\in \{30, 60, 90, 120\}$ min.

### Grammatical evolution

Structured Grammatical Evolution is based on Grammatical Evolution (GE)^19^, which is a variation of Genetic Programming that uses an evolutionary algorithm (EA). EAs are search and optimization algorithms that seek solutions by means of a process that emulates the process of genetic evolution. This process starts with an initial randomly generated population of individuals that represent solutions. These individuals are composed of a series of genes and alleles that represent the information they contain. A fitness value is assigned to each individual, depending on how close they are to the optimal solution. The individuals will undergo an evolutionary process of selection, crossover, and mutation. In the selection process, two individuals are chosen from the population based on some criteria. After that, they go through the crossover process, where new solutions are generated by interchanging the information in their genes. Finally, a mutation process is emulated on the population, introducing small random changes in the genes that represent the solution. This process is performed on the entire population to obtain a new population of children solutions. The whole process (selection, crossover, and mutation) is called a generation, and the evolution is repeated with the new population for a specified number of generations. At the end of the process, the best individual returned will represent the best solution found.

**Figure 1:**
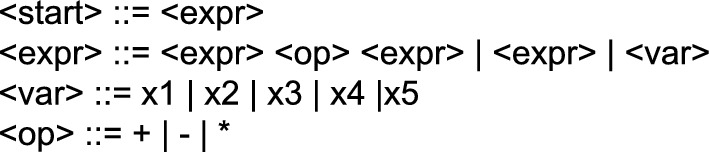
Example of a grammar.

GE uses this same schema to perform the optimization of the problem, but it specifies the structure of the individual following a set of rules, a.k.a. a grammar, and has to perform a specific process to determine the fitness of the individuals. As mentioned, the main feature of GE is the use of a grammar to perform the de-codification, mapping, between an individual’s genotype and phenotype. Figure 1 is an example of grammar in Backus-Naur form notation. The genotype will be a list of numbers, where each number is used to expand one non-terminal symbol of the grammar. Starting from the first rule in the grammar, the decodification process takes the first number in the individual and uses it to get the next production, composed of a set of non-terminal and terminal symbols. The terminals are added directly into the phenotype. For the non-terminal, it expands them in order, searching for the rule that corresponds to each one of them in the grammar and using the following number of the genotype to expand them as before. Since any number of the genotype can be used when expanding a non-terminal, the modulus operation is performed between the number in the genotype and the number of possible options in the grammar to expand the symbol. In this way, a valid option is always obtained.

GE can be tailored for any type of prediction problem, adding expert knowledge of the problem to the resulting phenotypes. In this way, it is able to generate different languages, by using different grammars, guiding the solutions in the search space. However, GE has a couple of known issues: (i) it has low locality, small changes in the genotype might produce big changes in the phenotype, therefore the mutation and crossover process might become disruptive, and (ii) redundancy is added by the modulus operator in the decoding process. In order to solve those issues, Lourenço et al. proposed a new approach called Structured Grammatical Evolution (SGE)^15^.

### Structured grammatical evolution

In SGE, the genotype has an additional structure; instead of a list of numbers, it is composed of a list of lists, one for each non-terminal of the grammar. It also ensures, through different methods, that the generated individuals are always valid, which is not always the case in the original GE implementation.

The decoding process begins with the first rule of grammar. Instead of directly using the first number in the genotype, it accesses the internal list corresponding to this non-terminal. From this list, it retrieves the first element and advances the index. With this number, it identifies the production defined by the first rule at the position indicated by the number. This process iterates for each symbol in the production. If the symbol is a non-terminal, it locates the corresponding internal list and retrieves the following unused number. This number determines the associated production, and the process repeats. If the symbol is a terminal, it is added to the phenotype, and the decoding continues with the following symbol from the production. Utilizing this internal structure in the genotype facilitates mutation and crossover operations, thereby addressing the issues mentioned with GE.

Initially, SGE employed static lists to encode individuals. Consequently, each internal list corresponding to a rule in the grammar was generated to its maximum length, even if not all expansions were utilized for decoding a specific individual. However, this approach becomes infeasible due to the potentially infinite references to recursive rules when dealing with a grammar containing recursion. To mitigate this, the grammar is transformed during execution by introducing an additional integer parameter defining the maximum recursive depth. This transformation involves duplicating the original rule to the specified depth and eliminating recursive productions, retaining only the non-recursive ones.

**Figure 2:**
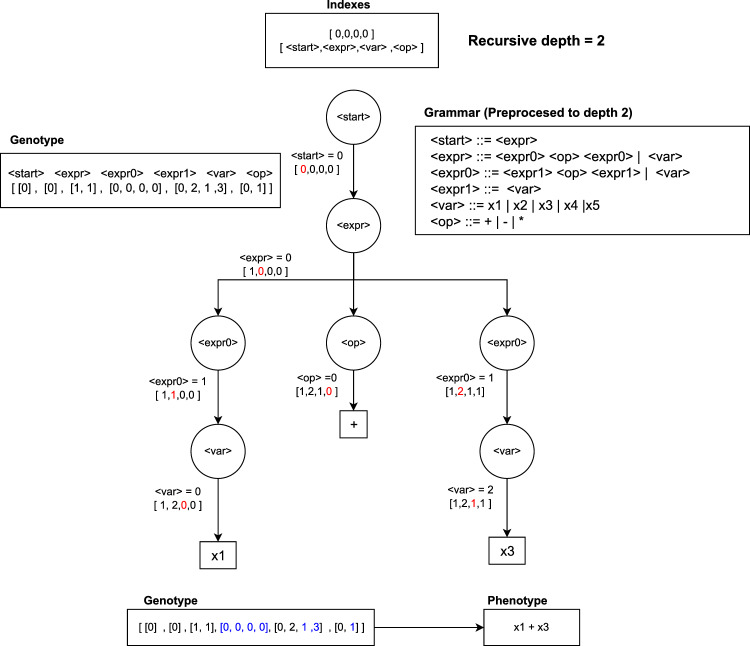
Example of a decoding process in SGE using a grammar with recursive depth of 2.

As an example, for depth 3, the following rule:
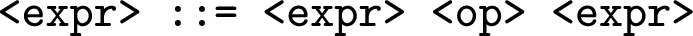


is transformed into:
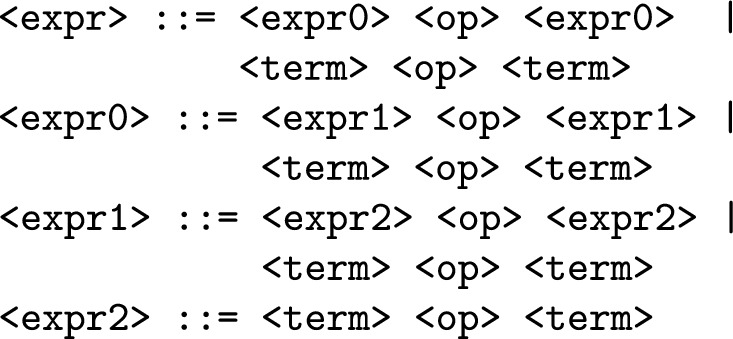


Figure 2 illustrates an example of SGE using a grammar with a recursive depth of 2. The recursive rule is transformed to a depth of two, and the length of each list is determined based on the maximum number of references. During the decoding process, the tree is generated starting with the symbol <start>, with subsequent productions determined by the corresponding numbers in the lists. The phenotype obtained after decoding is completed.

The original version of SGE addressed GE’s issues but introduced a new challenge by eliminating recursion and generating individuals up to a specified recursive depth. This results in potentially unused alleles, as depicted in Figure 2. However, these individuals still undergo mutation and crossover to maintain validity.

Dynamic SGE (DSGE), introduced by Lourenco et al.^20^, aimed to resolve this issue by dynamically adjusting individual growth during evolution. In DSGE, both genotypes and phenotypes are generated for the original population. If, during evolution, a phenotype becomes invalid, additional random elements are added to the genotype until validity is restored. Recursion in DSGE is managed by enforcing a maximum depth limit to prevent indefinite recursion. If the depth limit is exceeded, recursive rules are replaced with the shortest path to a terminal, selected randomly if multiple paths exist.

**Figure 3:**
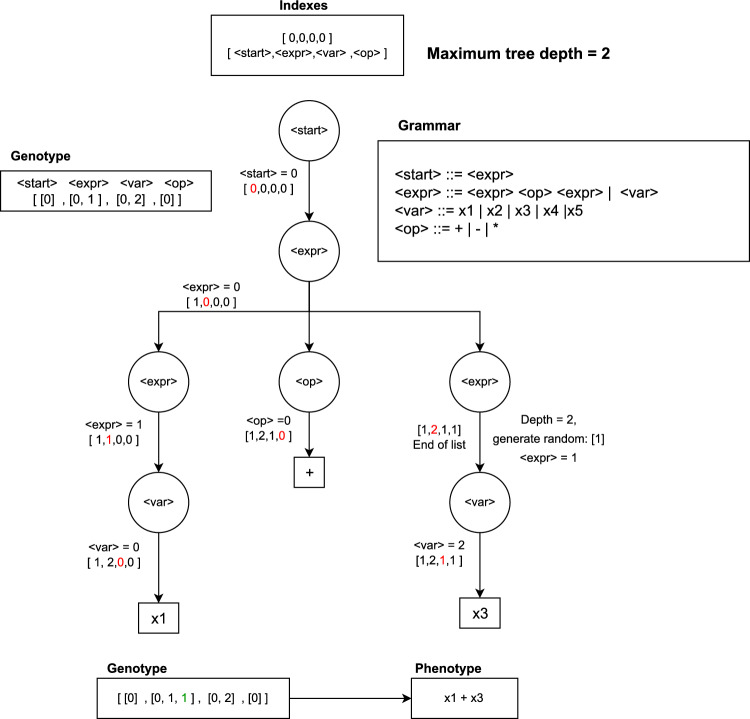
Example of a decoding process in DSGE using a grammar with recursive depth of 2.

Figure 3 illustrates a DSGE decoding process comparable to the SGE example. If the original genotype lacks sufficient genes for a non-terminal, random generation supplements the needed genes, maintaining the maximum tree depth. The addition of genes to ensure validity is denoted in green in the final genotype.

In controlling bloating (excessive growth of individuals), if a phenotype exceeds the maximum depth after evolution, DSGE generates non-recursive productions for subsequent expansions, ensuring adherence to the specified depth limit.

Let us see the details of the proposed grammar.

### Proposed grammar

**Figure 4:**
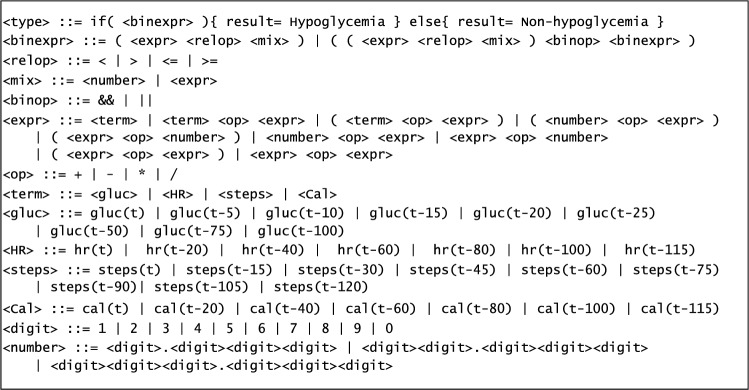
Grammar for forecasting.

As stated above, the genotype-to-phenotype translation is performed by means of a grammar, which determines the structure of the final models. Figure 4 presents the grammar we propose. This grammar does not make use of all of the input variables described previously. Instead, only a subset of variables is kept for each type (gluc, hr, steps, cal). The subsets have been generated by calculating the correlation values of the time series of each variable and keeping those that are less correlated to each other. In the case of gluc, we have decided to keep the last values read of the previous 25 min, in the 5-min intervals, as it adds information on the newest tendency before the time of prediction. The final model is composed of an $\mathrm {if-then-else}$ expression, whose condition determines if a sample is classified as hypoglycemia or non-hypoglycemia. The $\textrm{if}$ condition is made up of a combination of logical: and $\texttt { \& \& }$, or $\texttt {||}$, relative: $<, >, \le , \ge$, and numeric: $+, -, *, /$ operators, between the considered variables and constants. If the conditions are met, the observation is classified as hypoglycemia; if not, it is classified as non-hypoglycemia.

### Fitness function

The challenge of designing automatic classifiers for unbalanced datasets using genetic programming and grammatical evolution has been explored in prior research^15,21^. Nyathi et al.^22^ introduced the concept of weighted accuracy (WA) as a fitness function. This metric, represented by Eq. (1), aims to optimize both accuracy and the $F_1$ score of predictions simultaneously. The maximum attainable value of WA is 1.

1 $$\begin{aligned} \begin{aligned} \text {WA} = { 0.50 }*\text {Accuracy} + { 0.50 }*F_{1} \end{aligned} \end{aligned}$$

*Accuracy* is the ratio of elements correctly classified in both classes, and $F_{1}$ is the harmonic mean of *Precision* and *Recall*, Eq. (2).

2 $$\begin{aligned} \begin{aligned} F_{1} = \frac{2*\textrm{Recall}*\textrm{Precision}}{\textrm{Recall}+\textrm{Precision}} \end{aligned} \end{aligned}$$

*Precision* is the fraction of elements that we are classifying correctly in the Hypoglycemia class from the total amount we have classified in this class, and *Recall* is the fraction of elements correctly classified from the total amount of elements in the Hypoglycemia class.

The fitness function used in the training phase is:

3 $$\begin{aligned} F_{\text {fitness}}=1 - \textrm{WA} \end{aligned}$$

### Personalized, general, and cluster models

**Figure 5:**
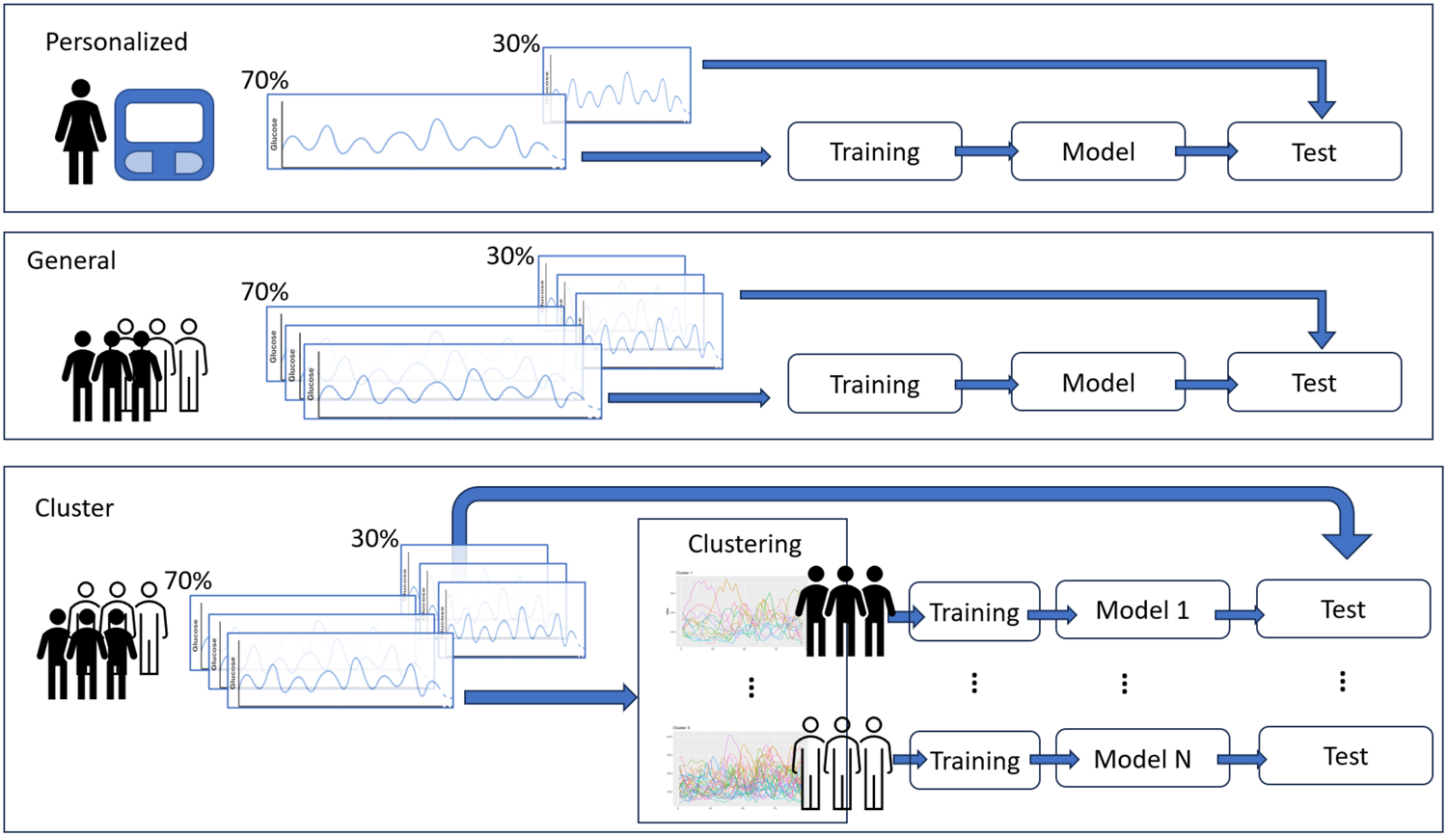
Personalized, General, and Cluster Models.

For each patient, we generate three different kinds of models to predict future hypoglycemic events, Fig. 5.

The first type are customized models for each patient, namely Personal Model (PM), which only uses data from the patient, so we train a specific model for each patient. In addition to PM, we also study the performance of other models that use data from several patients in the training phase, i.e., population-based models. A population-based model generates a single expression that is used for different patients in the prediction phase. We expect these models to work better than PM on patients with a reduced amount of data but worse than PM on patients with enough data for training.

We consider two types of population-based models. On the one hand, models that use the data from all the available patients to obtain an expression that will be used to predict. We will reference this type of model as a General Model (GM). On the other hand, we also construct models that use data from patients with similar features. To this aim, we first categorize the patients into different clusters and then generate an expression for each cluster. Each expression is used on all of the patients that belong to that cluster. We will reference this type of model as a Cluster Model (CM).

We use the K-Means algorithm to generate these clusters. K-Means algorithm creates *k* clusters with the variables of the data space, where *k* is defined by the user. It computes the distance between each centroid of the cluster and all the variables of an observation, each observation is set to belong to the cluster with the closest centroid. The centroids will move until the distance between them and all the observations that belong to each of them is the smallest possible. The clustering is performed on each patient characterized using the 4-D vector

4 $$\begin{aligned} \texttt {gluc\_var} = \left( \text {roc}(30), \text {roc}(60), \text {roc}(90), \text {roc}(120) \right) \end{aligned}$$

where $\text {roc}(w)$ is the average over time of the rate of change of $\texttt {gluc}(t)$ in $w \in \{30, 60,90,120\}$ min, Equation 5; i.e., the average speed at which a patient’s glucose changes over time.

5 $$\begin{aligned} \text {roc}(w) = \frac{1}{num\_samples}\cdot \sum _{t} \frac{\texttt {gluc}(t) - \texttt {gluc}(t-w)}{w} \end{aligned}$$

The metrics used to choose the number of clusters, *k*, will be the Elbow curve, Silhouette value, and the Davies-Boulding index.

### Data

**Table 1:** Characteristics of the participants: ID, Gender (M=Male; F=Female), Age (in years), BMI (Body mass index in $\hbox {kg/m}^{2}$), HbA1c (in %), Treatment (MDI: Multiples daily injections; CSII: Continuous subcutaneous insulin infusion); and years of evolution of T1DM.

ID	G	Age	BMI	HBA1c	Treatment	T1DM
HUPA001	F	56.3	22.8	8.2	CSII	15.5
HUPA002	M	48.6	23.8	7.1	CSII	36.5
HUPA003	F	43.4	18.7	7.3	CSII	12.5
HUPA004	M	41.2	27.2	7.8	CSII	8.5
HUPA005	F	20.9	22.6	6.9	CSII	39.5
HUPA006	M	22.1	24.6	7.8	CSII	13.5
HUPA007	M	37.6	30.6	6.6	CSII	10.1
HUPA010	F	41.9	19.0	6.0	CSII	15.2
HUPA011	F	35.0	23.9	7.8	CSII	27.3
HUPA014	F	50.0	25.4	8.5	MDI	12.9
HUPA015	F	43.1	22.3	6.4	MDI	11.2
HUPA016	F	29.9	26.3	6.5	CSII	20.1
HUPA017	F	26.3	22.2	8.2	MDI	24.2
HUPA018	F	32.3	20.5	7.2	CSII	25.6
HUPA019	M	18.0	24.7	7.1	MDI	7.6
HUPA020	M	45.7	25.4	9.7	MDI	13.5
HUPA021	F	48.6	24.4	7.5	MDI	2.2
HUPA022	M	59.6	24.2	6.7	CSII	14.6
HUPA023	M	22.9	18.5	7.7	MDI	0.8
HUPA024	M	47.9	26.6	8.3	MDI	35.9
HUPA025	M	38.1	29.6	7.0	CSII	20.3
HUPA026	F	61.8	29.4	7.2	MDI	21.5
HUPA027	M	26.4	22.2	7.0	MDI	23.7
HUPA028	F	21.2	21.9	6.1	MDI	2.0

We use data from 24 T1DM patients from the Hospital Universitario Príncipe de Asturias, Madrid (Spain). Participants signed an informed consent form, and the collection of the data was approved by the ethical committee of the Hospital in accordance with the Declaration of Helsinki guidelines^23^.

In the preprocessing phase of our study, it is important to note that data with missing values were subjected to a specific treatment. Instead of applying imputation techniques to complete the missing values, we decided to discard observations containing incomplete information. This approach was chosen to maintain the integrity and reliability of the data set, as imputation methods may introduce biases or inaccuracies. By choosing to eliminate cases with missing values, we aimed to ensure that subsequent analyses would be performed on a complete and homogeneous data set, thus minimizing the potential impact of missing data on our results, leaving the study of the influence of data imputation for subsequent work.

The main characteristics of the patients are in Table 1. It comprises 13 female and 11 male patients, ages ranging from 18 to 61 years old. The average age is (38.2±12.5) year (mean±std). The insulin treatment followed is either multiple daily injections of insulin for 11 patients or continuous subcutaneous insulin infusion for 13 participants. The years of evolution T1DM range from 0.8 to 39.5, with an average of (37.2 ± 18.3) year. This variable influences how much residual insulin the patient is still able to produce. 19 out of 24 patients have had T1DM for more than 10 years, so we can assume that endogenous insulin production is probably very low. The HBA1c value reflects the average blood glucose level for approximately the last two to three months^24^. As an average, HBA1c does not necessarily reflect how good the glucose control has been, so CGM is preferred by physicians for daily management of the disease. Nevertheless, it is used as a marker for high blood glucose in the diagnosis of diabetes or long-lasting issues in diabetic control. The recommended value for a T1DM patient is around 7%, approximately equivalent to an average of 154 mg/dL. The HBA1c values of the patients range from 6.0% (125 mg/dL) to 9.7% (232 mg/dL), with an average of (7.3±0.8)%. Most patients in the study maintain this value within the healthy range, although HUPA001, HUPA014, HUPA017, HUPA020, and HUPA024 have values higher than recommended, over 8.0%.

**Figure 6:**
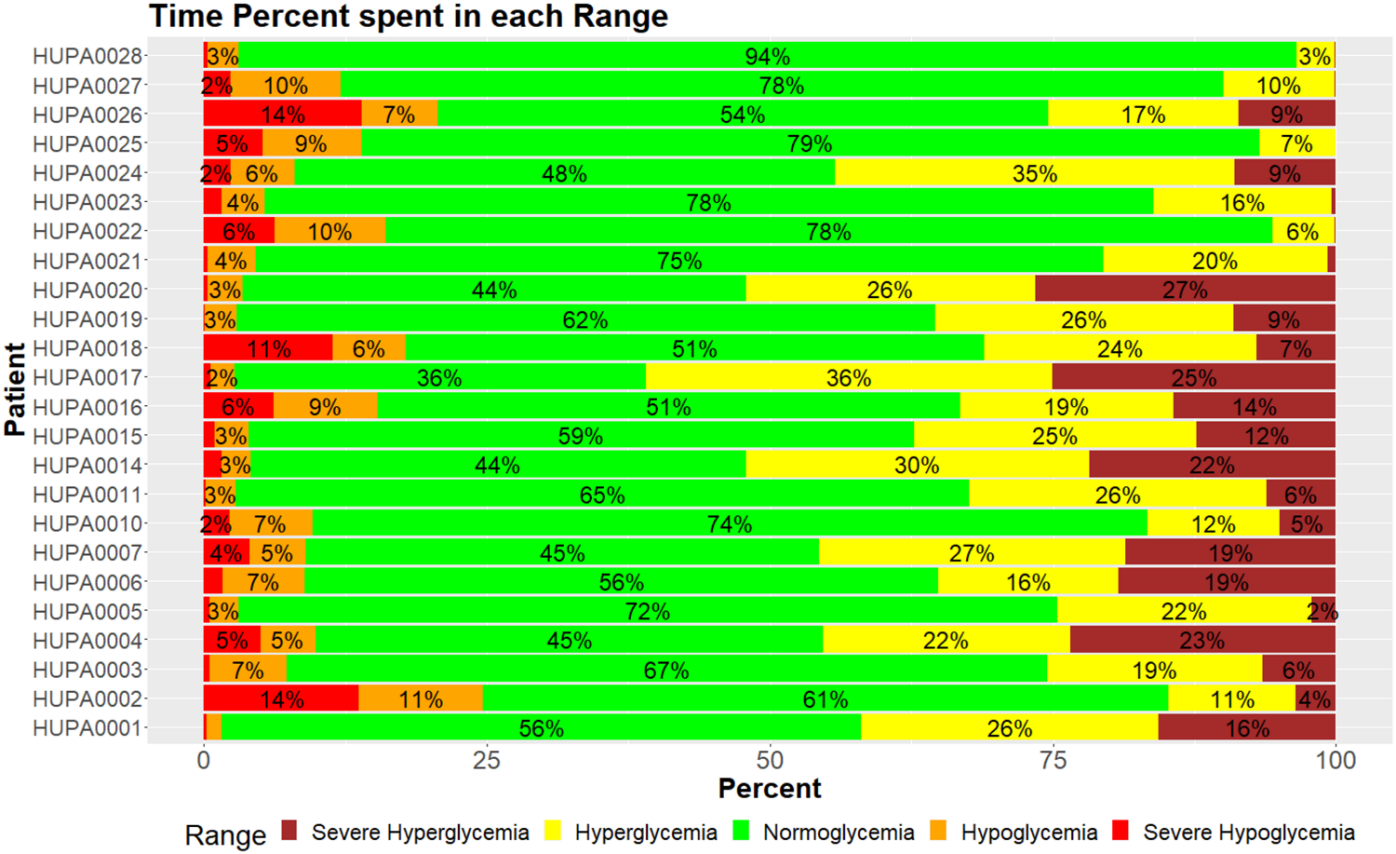
Blood glucose level distribution per patient where, Severe Hypoglycemia: < 54 mg/dL, hypoglycemia: < 70 mg/dL, Normoglycemia: $\ge$ 70 mg/dL $\cap \le$ 180 mg/dL, hyperglycemia: > 180 mg/dL and Severe Hyperglycemia: > 250 mg/dL.

Figure 6 displays the percentage of time that each patient spends in each glycemic interval. The time spent in hypoglycemia depends on each patient, with some spending less than 4% of the time and others more than 20%.

### Experimental setup

**Figure 7:**
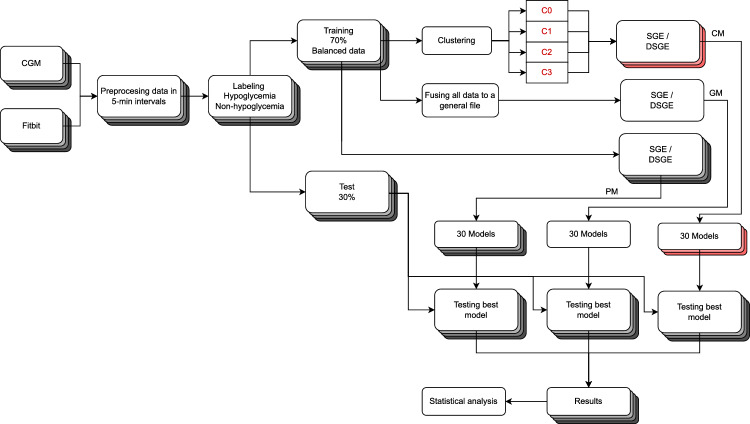
Overview experimental setup description.

Figure 7 shows a general overview of the experimentation workflow. First, we divide and label our data into two classes: hypoglycemia if the value we are trying to predict is lower than 70 mg/dL, or non-hypoglycemia if it is above it. Then, the data of the participants is divided into training and test, 70% and 30%, respectively. The sampling is randomly chosen to maintain the same proportion of hypoglycemic events in the training and test datasets. Afterward, the training dataset was balanced, performing random undersampling^25,26^, to obtain 50% of hypoglycemic and 50% of non-hypoglycemic values. This is necessary as the fitness function uses the accuracy metric, and the data is highly unbalanced. The data distribution depends on the patient as described in Figure 6. We ran the training 30 times and obtained a set of 30 best-fitness expressions. With them, we performed the test on each patient’s data.

**Table 2:** Experimental setup for SGE and DSGE.

Parameter	SGE	DSGE
Runs	30	
Replacement	The best between parents & offspring	
Selection	Tournament pressure 2	
Depth	Recursive: 3	Tree: 7
Population size	100	100
Generations (t+30 and t+60)	500	500
Generations (t+90 and t+120)	800	800
Mutation	Integer-Flip	
Mutation probability	0.05	0.05
Crossover probability	0.7	0.7
Crossover	Structured	Uniform
Crossover change probability	0.6	0.2

We investigated the performance of both algorithms, SGE and DSGE. Table 2 describes the setup of both algorithms on the search of the three kinds of models, PM, CM, and GM. The parameters have been set based on experimental tests, trying to be as fair as possible on both algorithms. The depth values have been set to be approximately equal and small enough to allow the results to be explainable.

For our experiments, we utilized two programming languages: Java 1.8 and Python 3.10. The machine learning and undersampling techniques were implemented using Scikit-learn (version 1.1.2). Regarding the machine characteristics, our system is equipped with an Intel(R) Core(TM) i7-7700 CPU @ 3.60GHz and 16.0 GB of RAM (15.8 GB usable).

### Ethical approval

Data have been specifically collected for this study. The collection of the data and all the protocols were approved by the ethical committee of the *Hospital Universitario Principe de Asturias’s* Clinical Research Ethics Committee. They are included in a database created for the project F11/2018. All the experiments were performed in accordance with the Declaration of Helsinki guidelines.

## Results

### Prediction results

This subsection shows the results obtained for the four time horizons: 30, 60, 90, and 120 min. We will also study the expressions obtained and the frequency of appearance of the variables in them.

**Figure 8:**
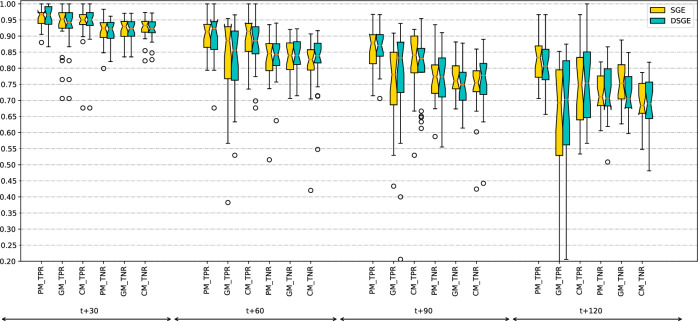
Boxplot comparison of TPR and TNR for all prediction horizons. DSGE results are colored in green and SGE in yellow. Three types of models are compared: PM, GM  and CM. A hypoglycemia event is considered a positive, while a non-hypoglycemia is a negative.

The area under the ROC curve (AUROC) is a widely used metric for evaluating classifier performance. However, this metric requires the classifier to generate a continuous output ranging from 0 to 1. In our specific scenario, the output of grammatical evolution consists of conditional expressions yielding binary results (0 or 1), which makes using AUROC impossible. Consequently, we will adopt an alternative approach to analyze the results in this paper, considering two complementary methods. On the one hand, we measure how well our models perform using the True Positive Rate (TPR) and True Negative Rate (TNR), defined in Eqs. (6) and (7) respectively. TPR corresponds to the fraction of elements correctly classified as hypoglycemia from all the hypoglycemic values and TNR to the fraction of elements correctly classified as non-hypoglycemia from all the non-hypoglycemic values. Figure 8 represents the results in the form of boxplots of TPR and TNR for all prediction horizons and both algorithms, DSGE and SGE.

6 $$\begin{aligned} \text {TPR}= & {} \frac{\textrm{truePositives}}{\textrm{positives}} \end{aligned}$$

7 $$\begin{aligned} \text {TNR}= & {} \frac{\textrm{trueNegatives}}{\textrm{negatives}} \end{aligned}$$

**Figure 9:**
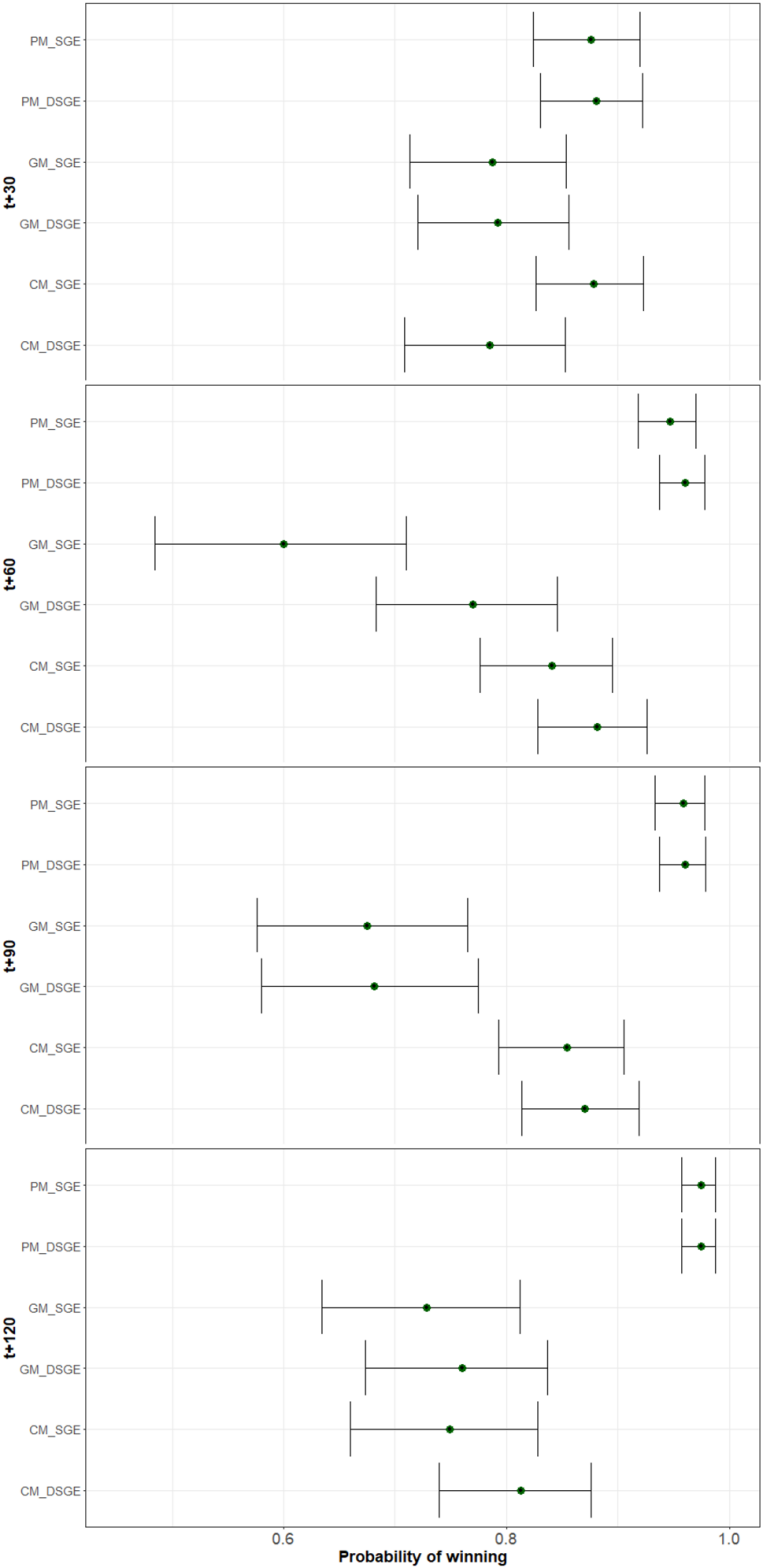
Bayesian ACC (average coverage criterion) for both SGE and DSGE.

On the other hand, we rank the performance of the models using two techniques based on Bayesian models for comparing the sampling techniques^27^. The Bayesian models are based on the Plackett-Luce distribution over historical values and time horizons^28^. We use a significance level $\alpha$ = 0.05 , with 20 Monte Carlo chains and 4000 simulations. Figure 9 contains the results of the probability of a model being the best (i.e., probability of winning) for all time horizons.

For the 30-minute prediction horizon, the results range around 0.95 for TPR and 0.92 for TNR for both algorithms in all the models considered. The differences among PM, GM, and CM models are very small. The PM has the best results on 11 patients, the GM on four, and the CM on nine patients.

Regarding the statistical analysis, the probability of winning is the highest for both PMs and the CM of SGE (a.k.a. CM_SGE). The other three models have less probability of winning, but the difference is not significant for any of the models, as the probability bands overlap.

For the 60-minute prediction horizon, the results obtained are around 0.85 to 0.90 for TPR and 0.82 to 0.85 for TNR. The PM obtains better results in the TPR in comparison to the GMand is very similar to the CM. In terms of the TNR, all three models obtain akin plots. Compared to the 30-minute prediction, on average, the classification results are lower, and the interquartile range (IQR) of the boxplots is wider. It represents that the results are more dispersed. The outliers obtained are more prevalent as their values are also lower, especially in the GM.

The PM obtains the best results on 19 patients, the GM on one, and the CM on four patients. This is a clear decrease in the win rate of the GM and CM, compared to the previous time horizon.

In the statistical analysis for the 60-minute prediction, results show a clearer difference between the different types of models in comparison to 30-minute models. The PM yields better results than the CM, and the GM. The probability bars for the GM and PM never overlap, meaning that there exists a statistical significance between the two. The CM the bars overlap with the GM of DSGE, likewise the DSGE CM overlaps briefly with the SGE PM, making the difference not significant. The graph also shows that between each model type, the DSGE models yield a higher probability than the SGE ones; however, there is no significant difference between the two.

The 90-minute prediction attains values of around 0.80 to 0.85 TPR and 0.75 to 0.8 TNR, although the values depend a lot on the model. In this time horizon, the gap between the TPR and TNR is much wider, especially in the PM  which has the best results in terms of TPR but whose TNR is very similar to the other two models. The GM has the widest IQR out of all three models in the TPR. This means that the distribution of results has a wide range of values, which indicates that the general solutions give widely different results for different patients. The CM works slightly better than the GM, and has a higher TPR average, but is still lower than the PM in almost all patients. The outlier values found are comparable to those for 60 min. In this time horizon, the PM gives the best results in 21 patients, and the CM in the other three patients.

The difference in the probability of winning between the models increases for the 90-minute prediction. There is a statistical difference in the performance between models, from which they get that the PM is better than the CM, and those better than the GM. As before, there is no significant difference between SGE and DSGE, and the probability bars are very similar for both algorithms.

The 120-minute prediction is the one with the lowest average TPR and TNR. The TPR is very different for the three models, with an average of 0.82 for the PM, 0.75 for the CM, and 0.70 for the GM. In terms of the TNR, the values are much more similar, around 0.72, although the CM is a couple of points below 0.70.

The performance loss obtained when the time horizon rises can be a result of a model generated without considering when it is applied. There is no distinction between the time of day the prediction is performed or the state (before or after a meal, sleeping, etc.). Therefore, after 120 min, the state of the glucose could have gone through a lot of changes. In this time horizon, we also observe the biggest difference between the GM and PM models in terms of TPR. The CM is better than the GM in the TPR, especially on patients with much worse results on the GMbut worse on the TNR. The GM and CM are only the best in one case each; for the other 22 patients, the PM gives better results.

Another observation is that the CM does not yield as good results as expected if we compare them to the GM, especially for the 120-minute time horizon. This indicates that the method to generate the clusters, which is based on dividing the patients by their glucose variability, is not particularly appropriate and should be reconsidered.

In conclusion, the results show that the GM and CM yield worse results than the PM in terms of the TPR, and the longer the time horizon, the wider the gap. This observation is not true for the TNR, where the results are very similar for the three models. It also reveals that the TNR of the models is, on average, lower than the TPR, but the interquartile range (IQR) of the results is narrower. A possible explanation for this behavior is the fact that we have fewer instances of hypoglycemia on test in comparison to those of non-hypoglycemia. The difference in the algorithm, SGE versus DSGE, is very small and not statistically significant in any case.

In terms of the statistical analysis between methods, we conclude, firstly, that the difference in the probability of winning between the PM and the CM has increased. In turn, it has decreased between the CM and the GM, with the CM being slightly better than the GM. Like before, both algorithms show no significant difference between each other; however, DSGE has a slightly higher probability of winning on both the GM and CM.

### Comparison with other ML algorithms

We have considered eleven ML algorithms to compare their performance with our proposal. This comparison demonstrates what our technique is able to achieve in terms of accuracy with different types of algorithms and different levels of interpretability. The algorithms used and their hyperparameters are shown in Table 3. They are Gradient Boosting (GB), Logistic Regression (LR), Random Forest Classifier (RF), Decision Tree Classifier, AdaBoost Classifier, Bagging Classifier, Extra Trees Classifier, SGDC Classifier, Multi-Level Perceptron Classifier (MLP) and Support Vector Machine (SVM).

**Figure 10:**
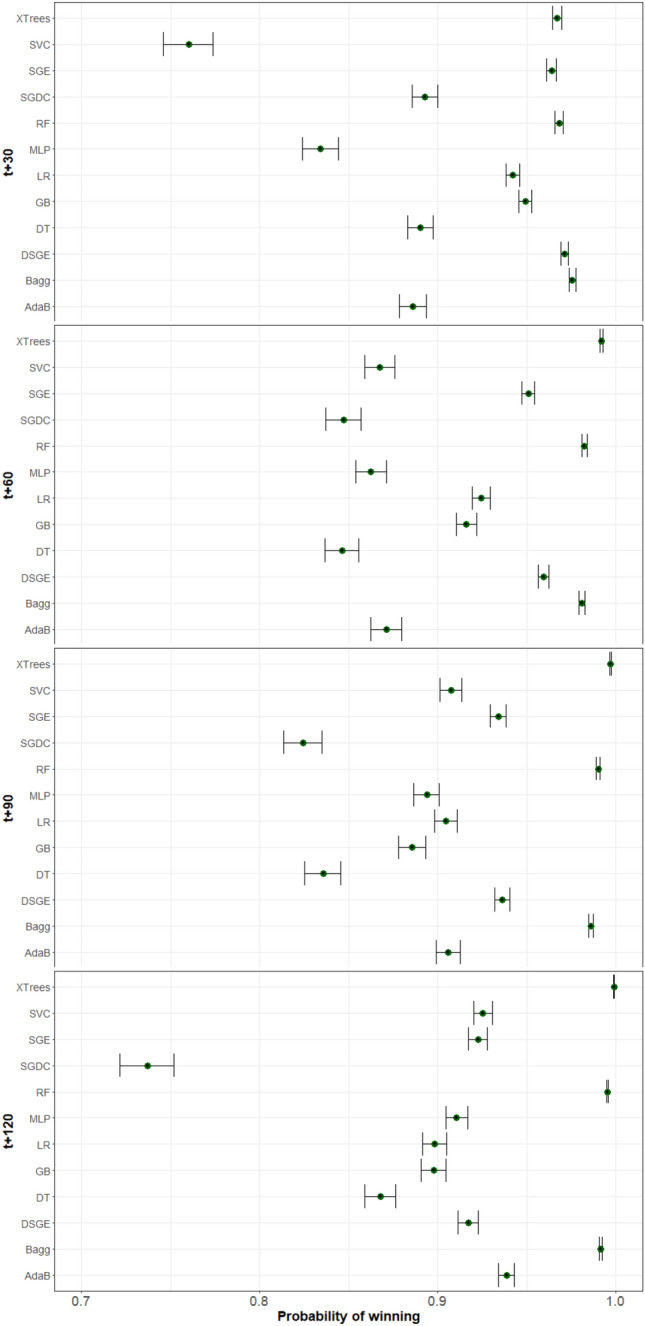
Bayesian comparisons for all time horizons of 30 subsets. The techniques with the bars at the right of the figure have a higher probability of being better than those on the left.

The study has been performed by generating 30 different subsets of training and test, and training a GM for each algorithm. The resulting models have been tested on the data for each patient. Figure 10 contains the statistical study of the results for each time horizon.

**Table 3:** Hyperparameters for Machine Learning Techniques.

Technique	Hyperparameters
Gradient boosting	n_estimators: 50-300 learning_rate: 0.01-0.1 max_depth: 3-8 min_samples_split: 2-20 min_samples_leaf: 1-10
Logistic regression	C: 0.01-10 penalty: {’l2’} solver: {’lbfgs’}
Random forest classifier	n_estimators: 50-300 max_depth: 3-8 min_samples_split: 2-20 min_samples_leaf: 1-10
Decision tree classifier	max_depth: 3-8 min_samples_split: 2-20 min_samples_leaf: 1-10
AdaBoost classifier	n_estimators: 50-300 learning_rate: 0.01-1.0 base_estimator: DecisionTreeClassifier(max_depth=1)
Bagging classifier	base_estimator: DecisionTreeClassifier(max_depth=3) n_estimators: 50-300
Extra trees classifier	n_estimators: 50-300 max_depth: 3-8 min_samples_split: 2-20 min_samples_leaf: 1-10
SGDC classifier	loss: {’hinge’, ’log’, ’modified_huber’} alpha: 0.0001-0.01 penalty: {’l1’, ’l2’}
Multi-level perceptron classifier	hidden_layer_sizes: (100,) activation: {’tanh’, ’relu’} solver: {’adam’, ’sgd’} alpha: 0.0001-0.01
Support vector machine	C: 0.1-100 kernel: { ’rbf’} gamma: { ’auto’}

The graph shows a clear difference between the performance of the algorithms and the time horizons considered. On the shorter time horizons, we observe that our proposals (DSGE and SGE) have a high probability of winning and that the probability diminishes on the longer time horizons. For the longer time horizons, the ensemble methods XTrees, RF, and Bagg yield the best results, with statistical significance to win. The methods proposed yield average results on the comparison.

From this analysis, we conclude that the difficulty of the problem in the longest time horizons makes the more complex ensemble methods perform better at the expense of the explainability of the resulting model. Our proposals obtain better or equal results to the other algorithms, making it a good contender in terms of both explainability and accuracy.

### Cluster results

As mentioned previously, we are interested in studying whether we can obtain a single model that works well on different patients. This model can be created with the data from all the patients or by using data from a subset of patients that might share a few characteristics. We use the implementation of the Sklearn Python library to perform the clustering.

From the training data of the 24 patients and using the variables described previously, we generate the Elbow curve, Silhouette value, and the Davies-Boulding index for $k \in [2,7]$ to determine the optimal value for *k*. From these metrics, we have chosen $k=4$, as it yields the least amount of clusters with the best results on all the metrics considered. The final clusters with the patient’s ID are:

*Cluster*0 : [27, 22, 25]

*Cluster*1 : [2, 3, 5, 6, 10, 11, 14, 16, 18, 21, 23, 24, 26]

*Cluster*2 : [1, 4, 7, 15, 19]

*Cluster*3 : [17, 20, 28]

From this division, we can see that *Cluster*1 has significantly more people than the other three clusters obtained. That should necessarily impact the results values of those patients compared to the other clusters. Cluster0 includes three persons with a high time in range (time in values of glucose [70, 180] mg/dL); however, we also find participants with high time in range values in other clusters, such as HUPA028.

Although the clustering analysis is not the focus of the paper, a more in-depth study of other forms of clustering and the inclusion of other variability metrics^29^ would be recommended for future work.

## Discussion

### Interpretability of the results

While developing machine learning models in healthcare, prioritizing interpretability is paramount^30^. However, the landscape of interpretability is intricate, with ongoing debates on its definition and measurement. Critical factors associated with interpretability include complexity, transparency, and the capacity for simulation, each viewed through the lens of different evaluators. Drawing inspiration from the seminal works of Lipton (2018)^31^ and Belle (2021)^32^, we delve into three crucial dimensions for analyzing the interpretability of our model: simulatability, decomposability, and algorithmic transparency.

**Figure 11:**
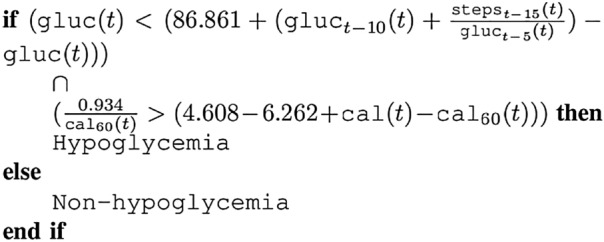
Example of an expression obtained using our technique.

One of the main advantages of SGE and DSGE models is that they generate interpretable expressions, i.e., the expressions explicitly present the variables used and how they interact with each other. For the sake of clarity, we present as an example the resulting expression obtained for patient HUPA006 at the time horizon of 30 min for DSGE and study which elements it has focused on to perform the prediction. Figure 11 displays one example expression. It is composed of two conditions separated by an ”and” $(\cap )$ operator; they must both be true to classify a sample as hypoglycemia.

The first part of the expression can be rewritten as:

$$(2 \cdot \texttt {gluc}(t) - \texttt {gluc}_{t-10}(t) -\frac{\texttt {steps}_{t-15}(t)}{\texttt {gluc}_{t-5}(t)} < 86.861$$

It subtracts the glucose value 10 min prior to the time of prediction, and the division between the steps 15 min prior and the glucose value 5 min prior, to two times the current glucose value. This difference must be less than 86.861 to consider the observation hypoglycemia. The constant 86.861 is 16 points above the hypoglycemia limit of 70 mg/dL. If the value of the glucose 10 min prior to the time of prediction is higher than the current glucose, the difference between them will be negative, and the glucose value is decreasing, which in turn will lower their side of the equation towards the condition being true. The fractional element that also subtracts is composed of step values as the numerator and the amount of glucose as the denominator. The division is always positive or zero, so it can only lower this side of the equation; however, the amount will be very small unless the steps performed at 15 min prior to prediction are very high or the glucose value is very low. This conforms to the idea that performing exercise lowers blood glucose values, although, this is not always the case, as is shown in the second part of the condition.

The second part of the condition can be rewritten as:

$$( 1.654 >\texttt {cal}(t) - [ \texttt {cal}_{60}(t) + \frac{0.934}{\texttt {cal}_{60}(t)}])$$

This part states that an event is classified as hypoglycemia if the difference between the current calories burned and the calories burned 60 min ago plus the inverse of the calories burned 60 min ago, is lower than 1.654. This states that if the calories burned at the time of prediction decrease or stay within the specified range, in comparison to the calories burned 60 min in the past, then a hypoglycemic event is possible. However, if the burned calories increase by the specified amount, a hypoglycemia prediction is not performed. This is a very interesting condition since it represents that in training, for patient HUPA006, if there was a sudden increase in the calories burned just before the time of prediction then a hypoglycemic event does not occur. This phenomenon could indicate, for example, the patient waking up, an increment in movement, or stress levels, but it is a condition very specific to the patient.

As observed from the example, the models generated find non-trivial patterns in patient data, which can then be eventually adapted and studied.

From the resulting models, we have extracted the variables that each expression considers to perform the classification and determined that gluc(t) is the most used variable in the models, appearing in 95% of all solutions. This behavior is expected as it is the last glucose value we have before performing the prediction. The rest of the variables appear much less in comparison, between 20% and 35% of the solutions. Since the expressions are limited by size, not all variables can appear, so we get smaller percentages for the other input variables. The steps variables appear slightly less than the other two exercise adjacent variables; hr, and cal. This behavior indicates that the number of steps is not as important for the models compared to the other types; this might be due to the fact that the calories burned are computed, among other things, using the number of steps, and this variable is more informative. In addition, the variables of each type with the highest percentage are those from the time of prediction.

To sum up the characteristics of our methodology in the three dimensions of interpretability:

- Simulatability: This dimension revolves around the model’s ability to be simulated by a human, with simplicity and compactness as defining attributes. Our models, composed of if-then-else rules, are inherently simulatable, as we have seen. In addition, they can assist clinicians in understanding the dynamics of blood glucose behavior.
- Algorithmic Transparency: Focusing on understanding the procedural steps employed by the model, this dimension is crucial for unraveling the model’s outputs. Models employing clear and understandable procedures, such as similarity-based classifiers (like K-means), showcase algorithmic transparency. Conversely, complex models like neural networks, constructing elusive loss functions, demand mathematical analysis for insight into their procedural steps. The different components of our methodology, K-means clustering, grammatical evolution, and the final model, which is composed of if-then-else statements, exhibit this feature.
- Decomposability: This dimension involves breaking down the model into interpretable components, including inputs, parameters, and computations, and explaining each facet. Our approach exhibits this dimension as the final model, and all the steps in our methodology are interpretable.

### Limitations

Although our database consists of 24 individuals (compared to 12 individuals in the Ohio T1DM dataset^33^, one of the most used datasets in the bibliography^34^), the pool of people with diabetes is still relatively small, and this may affect the generalisability of our results.

Our analysis does not differentiate between CSII and MDI treatments. This lack of distinction may overlook potential variations in treatment response, limiting the granularity of our conclusions, and should be addressed in the future.

Clustering was performed solely based on the rate-of-change variable. Given the unsatisfactory prediction results from this approach, we recognized the necessity to explore alternative techniques and incorporate additional variables for a more comprehensive analysis.

Our classifier operates as a ”hard decision binary classifier,” lacking the ability to provide probability estimations. Consequently, we cannot employ the widely used AUC-ROC (Area Under the Curve-Receiver Operating Characteristics) metric, a common practice in other binary classifiers. While our TPR and TNR results show competitive records in short prediction horizons, the absence of AUC-ROC analysis limits the comprehensive evaluation of our model.

## Conclusion

In this paper, we showed the performance of two versions of Structured Grammatical Evolution (SGE and DSGE) in the context of hypoglycemia prediction for four time horizons (30, 60, 90, and 120 min). We have tested different population-based models and observed how each patient reacts to generalizing in such a way. The best results are obtained with personalized models for the TPR. However, the GM can yield similar TPR results on short time horizons and equal or better results for the TNR. When performing the test on each patient, we observed that some follow widely different patterns. This effect can be helped using clusters, and it is recommended when the number of patients increases since the more patients we add, the more probable that some of them are not taken into account in the final model obtained. Even so, general models are only recommended for more extended time horizons if we can find a set of patients whose glucose development is very similar or if more data is needed to train a personalized model.

As previously stated, the resulting models obtained are if-then-else statements with conditions including input variables and some constants. As the models obtained comprise numeric, relational, and logical operations between variables, they can be explained, studied, edited, and retested. This freedom opens many possibilities for tweaking reasonable solutions after being obtained to fit the problem better or to study cases where the model has failed and why it has failed. The models can be trained and consulted through mobile and online applications.

In the future, we can test the efficiency of the algorithm on different class divisions and use other variables, such as insulin and carbohydrates, from which we can predict future glucose states by using more information than the current glucose state, adding these variables. On the other hand, other clustering techniques and variables should be considered to increase the efficiency of the models. We will study whether the clustering should be applied to all patients or only a subset.

In terms of class division, we should consider having different types of divisions so that the 70 mg/dL is not a hard limit since the CGM sensors have a certain error, and different individuals use different thresholds to perform their glucose control.

## Data Availability

Our data is available upon reasonable request. Please contact the corresponding author to request access to the data.
